# Scavenger receptor class B, type I (*Scarb1*) deficiency promotes osteoblastogenesis but stunts terminal osteocyte differentiation

**DOI:** 10.14814/phy2.12117

**Published:** 2014-10-03

**Authors:** Corine Martineau, Olha Kevorkova, Louise Brissette, Robert Moreau

**Affiliations:** 1 Laboratoire du Métabolisme Osseux, BioMed, Département des Sciences Biologiques, Université du Québec à Montréal, Montréal, H3C 3P8, Quebec, Canada; 2 Laboratoire du Métabolisme des Lipoprotéines, Département des Sciences Biologiques, BioMed, Université du Québec à Montréal, Montréal, H3C 3P8, Quebec, Canada

**Keywords:** Differentiation, HDL, MSC, osteoblast, osteocyte, proliferation, SR‐BI, Wnt pathway

## Abstract

Scavenger receptor class B type I (SR‐BI), the *Scarb1* gene product, is a high‐density lipoprotein (HDL) receptor which was shown to influence bone metabolism. Its absence in mice is associated with alterations of the glucocorticoid/adrenocorticotropic hormone axis, and translated in high bone mass and enhanced bone formation. Since the cellular alterations underlying the enhanced bone formation remain unknown, we investigated *Scarb1*‐deficient marrow stromal cells (MSC) behavior in vitro. No difference in HDL_3_, cholesteryl ester (CE) or estradiol (E) association/binding was measured between *Scarb1*‐null and wild‐type (WT) cells. *Scarb1* genic expression was down‐regulated twofold following osteogenic treatment. Neither WT nor null cell proliferation was influenced by HDL_3_ exposure whereas this condition decreased genic expression of osteoblastic marker *osterix* (*Sp7*), and osteocyte markers sclerostin (*Sost*) and dentin matrix protein 1 (*Dmp1*) independently of genotype. *Sost* and *Dmp1* basal expression in null cells was 40% and 50% that of WT cells; accordingly, osteocyte density was 20% lower in vertebrae from *Scarb1*‐null mice. Genic expression of co‐receptors for Wnt signaling, namely *LDL‐related protein* (*Lrp*) *5* and *Lrp8*, was increased, respectively, by two‐ and threefold, and of transcription target‐genes *axis inhibition protein 2* (*Axin2*) and *lymphoid enhancer‐binding factor 1* (*Lef1*) over threefold. Gene expression of Wnt signaling agonist *Wnt5a* and of the antagonist *dickkopfs‐related protein 1* (*Dkk1*) were found to be increased 10‐ to 20‐fold in null MSC. These data suggest alterations of Wnt pathways in *Scarb1*‐deficient MSC potentially explaining their enhanced function, hence contributing to the high bone mass observed in these mice.

## Abstract (precis)

Scarb1‐deficiency translated in high bone mass and enhanced bone formation. We investigated Scarb1‐deficient marrow stromal cells (MSC) behavior in vitro. Our results suggest alterations of Wnt pathways in Scarb1‐deficient MSC potentially explaining their enhanced function, hence contributing to the high bone mass observed in these mice.

## Introduction

Scavenger receptor class B, type I (SR‐BI), the protein product of the *Scarb1* gene, is a high affinity high‐density lipoprotein (HDL) receptor (Acton et al. 1996) known for its role in the process of reverse cholesterol transport (RCT), defined as, the transport of cholesterol from peripheral tissues toward the liver for excretion (Zhang et al. 2005). RCT implies the cholesterol efflux from cells to HDL and the selective uptake of HDL‐associated cholesteryl esters (HDL‐CE) by hepatic SR‐BI and therefore, this receptor's functions are considered beneficial to the cardiovascular system (Mineo and Shaul 2012). Its absence correlates with impaired HDL cholesterol metabolism (Rigotti et al. 1997) and reduced HDL‐CE selective uptake (Brodeur et al. 2005). In accordance, *Scarb1*‐null mice display increased HDL‐associated cholesterol which agrees with its role in HDL metabolism (Rigotti et al. 1997). The process of HDL‐CE selective uptake is also preponderant in steroidogenic organs, such as ovaries and adrenal glands, where it supplies cholesterol molecules for steroid synthesis (Rigotti et al. 2003; Hoekstra et al. 2009). Additional functions of SR‐BI include selective uptake of other HDL‐associated lipophilic compounds by cells, such as estradiol (Badeau et al. 2007) and vitamin E (Mardones et al. 2002). Since SR‐BI has been shown to be expressed in macrophages (Hirano et al. 1999), and to bind and mediate internalization of oxidized low‐density lipoproteins (OxLDL) (Gillotte‐Taylor et al. 2001), it has been postulated that OxLDL uptake accounts for macrophage foam cell formation in atherosclerotic lesions. Although its role in the liver and steroidogenic tissues is well established, its functions in peripheral tissues are not clear. In enterocytes, SR‐BI is thought to promote cholesterol absorption (Altmann et al. 2002; Levy et al. 2007). In adipocytes, this receptor is associated with HDL clearance and subsequent efflux of adipose‐stored cholesterol to HDL, contributing to HDL cholesterol homeostasis (Yvan‐Charvet et al. 2007; Zhang et al. 2010).

We have reported expression of SR‐BI and selective uptake of HDL‐CE and estradiol by osteoblasts (Brodeur et al. 2008a). These cells contribute to the remodeling of bone tissue which relies on the activities of bone‐forming osteoblasts and bone‐resorbing osteoclasts, to maintain bone mass (Clarke 2008). We recently reported that *Scarb1*‐null mice show high femoral bone mass associated with enhanced bone formation (Martineau et al. 2014), suggesting that SR‐BI contributes to the regulation of bone metabolism. Since *Scarb1*‐null mice show high HDL‐associated cholesterol levels (Rigotti et al. 1997; Martineau et al. 2014), whether the high plasma HDL‐associated cholesterol levels observed in these mice contribute to the enhanced bone formation and high bone mass remain unknown. Studies focusing on the relationship between HDL and bone have yielded conflicting results; some associate higher plasma HDL levels to higher bone mass, while others have found no or inverse correlations (reviewed in Ackert‐Bicknell 2012). Interestingly, we have evidenced that selective uptake levels of HDL‐CE and HDL‐associated estradiol were similar between mesenchymal stromal cells (MSC) from wild‐type (WT) and *Scarb1*‐null mice (Martineau et al. 2014), suggesting that its contribution to this process is not its main role in these cells. On the other hand, intrinsic alterations of MSC functions from *Scarb1*‐null mice were evidenced in vitro, globally showing enhanced bone‐forming potential such as increased proliferation rate, higher alkaline phosphatase activity, enhanced matrix mineralization, and higher genic expression of the osteoblastic transcription factor *Sp7* (Martineau et al. 2014).

Of interest, we measured lower caveolin‐1 (*Cav1*) expression in MSC from *Scarb1*‐null mice (Martineau et al. 2014). Caveolin‐1 is a membrane protein whose expression is sufficient to generate the formation of flask‐shaped membrane structures termed caveolae (Parton et al. 2006). Caveolae are involved in several cellular pathways, either repressing or enhancing their signal through interactions with different receptors and intermediates (Razani et al. 2002; Cohen et al. 2004). Because of its importance in numerous signaling pathways, disruption of *Cav1* expression is expected to cause cellular disorders; for example, it is known to repress cyclin D1 expression and to keep cells in a quiescent state (Hulit et al. 2000; Galbiati et al. 2001). Although the exact mechanisms remain to explore, caveolin‐1 is also reported to regulate human MSC proliferation and differentiation toward an osteoblastic phenotype (Baker et al. 2012). Since similar alterations in *Scarb1*‐null mouse MSC were previously reported (Martineau et al. 2014), similar mechanisms may be at play. Of note, some receptors of canonical Wnt signaling, a pathway involved in osteoblastogenesis (Boudin et al. 2013), have been localized in caveolae, such as LRP6 (Yamamoto et al. 2006) and LRP8 (Riddell et al. 2001). The aims of this study were to further document the impact of *Scarb1* deficiency on MSC functions in vitro.

## Material and Methods

### Experimental animals

*Scarb1‐*null (−/−) mice on a C57BL/6 background were purchased from Jackson laboratories (stock #003379; Bar Harbor, ME) and cross‐bred with WT C57BL/6 mice. Heterozygous (HZ) littermates (initial parental couples, P0) were bred to yield first generation (F1) WT and null mouse pairs; since null females show low fertility, higher reproduction rates were achieved by feeding *Scarb1*‐null females a 0.5% Probucol diet (Research Diets, New Brunswick, NJ) prior to mating (Miettinen et al. 2001). HZ couples were bred in parallel with WT and null couples through each generation to allow direct WT and null littermate comparison. Null pup yield from HZ pairs being below 20%, null mice of F10 to F12 generations from both HZ and null pairs were pooled together and compared to appropriately matched WT groups. All individuals were kept in a 12 h/12 h day/night cycle with free access to food and water unless specified otherwise. All animal protocols were performed according to the UQÀM Institutional Animal Care Committee (IACC #757).

### Culture of bone‐marrow stromal cells

Long bones from 2‐month‐old male and female mice hind limbs were harvested and sterilized in phosphate‐buffered saline (PBS) containing 200 U/mL penicillin, 200 *μ*g/mL streptamycin and 1% FungiZone (all from Invitrogen, Burlington, Ont., Canada). Epiphyses were cut off under sterile hood and marrow was flushed out. Bone‐marrow cells were suspended in alpha Minimum Essential Medium Eagle (*α*MEM; Invitrogen) supplemented with 100 U/mL penicillin‐100 *μ*g/mL streptamycin, l‐glutamine, 10% fetal bovine serum (North Bio, Toronto, Ontario, Canada), 25 *μ*g/mL l‐ascorbic acid (Sarstedt, Montreal, QC, Canada) and plated; bones from one mouse were used per 100 mm culture dish (Sigma‐Aldrich). The cells were left to adhere for 7 days, and then thoroughly washed with PBS to eliminate nonadherent cells. Adherent cells (hereafter referred to as marrow stromal cells – MSC) were left to reach confluence prior to harvest and experimentation. Cells harvested from each mouse were considered as an independent biological replicate; since cells from males and females showed the same profile, the data were pooled for statistical purposes.

### Preparation of lipoproteins

Lipoproteins were isolated from human plasma (Bioreclamation, Hicksville, NY). Before isolation, the plasma was supplemented with 0.01% EDTA, 0.02% sodium azide, and 10 *μ*mol/L phenylmethylsulfonylfluoride (PMSF) to prevent degradation. Human HDL_3_ (density of 1.125–1.21 g/mL) and LDL (density of 1.025–1.063 g/mL) were prepared by ultracentrifugation as described previously (Brissette et al. 1996; Brodeur et al. 2008a). HDL_3_ and LDL were dialyzed twice for 24 h to remove EDTA; LDL were further incubated with 5 *μ*mol/L CuSO_4_ for 20 h at 30°C as described previously (Lougheed and Steinbrecher 1996; Brodeur et al. 2008a). Oxidation was stopped by the addition of 100 *μ*mol/L EDTA and 40 *μ*mol/L butylated hydroxytoluene (BHT). Oxidized LDL particles (OxLDL) were dialyzed twice more and were concentrated to 10–15 mg/mL using Centriplus‐100 *μ*L‐ultrafiltration devices (Amicon, Oakville, Ontario, Canada).

### Association and binding assays

HDL_3_ were radiolabeled as described previously (Brissette et al. 1996; Brodeur et al. 2008a) either through 125‐iodination, with 1,2‐[^3^H]cholesteryl oleate (CO) (Roberts et al. 1985) or [2,4,6,7,16,17‐^3^H]estradiol (E_2_). The specific activity for each ligand ranged between 65,000 to 105,000 cpm/*μ*g protein for [^125^I]‐HDL_3_, 18,000 to 18,300 cpm/*μ*g protein for [^3^H]‐CO‐HDL_3_ and 5500 to 5800 cpm/*μ*g protein for [^3^H]‐E_2_‐HDL_3_. Cellular associations of ^125^I‐lipoprotein or [^3^H]CO‐ or [^3^H]E_2_‐HDL_3_ were conducted as previously described (Brodeur et al. 2008a; Martineau et al. 2014). Cellular binding of ^125^I‐lipoprotein was measured using the same lipoprotein concentrations as association experiments, but were rather incubated at 4°C for 2 h to insure no cell metabolic activity or ligand internalization. After the incubation, cells were washed twice with 1 mL PBS containing 0.2% BSA, followed by two washes with 1 mL PBS, and solubilized in 1.5 mL of 0.2N NaOH. Radioactivity counts in the homogenates were obtained with a cobra II *γ*‐counter (Canberra‐Packard) for ^125^I‐HDL_3_ determination and with a *β*‐counter (Wallack‐Fisher) for measurement of [^3^H]CO‐ or [^3^H]E_2_‐HDL_3_ content. The results are expressed in micrograms of lipoprotein protein per milligram of cellular protein. Cellular protein contents were determined by the Bradford method (Bradford 1976).

### MTT activity and proliferation assays

MTT activity was determined in 96‐well plates (Sarstedt) by microtiter tetrazolium assay after incubation. Briefly, MTT reagent was added to the medium at a final concentration of 0.5 mg/mL. Four hours later, formazan crystals generated by cellular reduction in the MTT reagent were dissolved in dimethyl sulfoxide (DMSO) for 30 min at 37°C and the absorbance was determined at 575 nm. Results are expressed as the relative MTT activity of treated versus control conditions.

### Osteogenic differentiation treatment

MSC were seeded in 6‐well plates at 50,000 cells/cm^2^ and cultured to confluence; monolayers were then treated 3 times a week with control (MEM supplemented with 10% FBS) or osteogenic medium (control medium supplemented with 50 *μ*g/mL ascorbic acid and 5 mmol/L glycerol‐2‐phosphate) for 3 weeks. After the treatment, differentiation was assessed by ALP and ARS staining as described elsewhere (Martineau et al. 2014) in half of the wells; the other wells were used to observe genic expression of *Scarb1*.

### Osteocyte density

Following CO_2_ euthanasia, lumbar vertebrae from 10‐week‐old mice of both genotypes were harvested, fixed overnight in 4% paraformaldehyde at 4°C, and processed to be paraffin‐embedded. Bones were decalcified for 14 days in 10% EDTA‐PBS prior to processing. Vertebrae were sectioned at a 10 *μ*m thickness with a HM360 rotary microtome (Thermofisher) and mounted on SuperFrost Plus glass slides (Thermofisher). Sections were heated flat at 60°C for 10 min prior to procedure; tissues were then deparaffinized and rehydrated. The sections were rinsed 3 × 5 min in PBS stained with DAPI (Invitrogen), mounted with ProLong (Invitrogen) and visualized with a FC1 Eclipse inverted fluorescence microscope (Nikon) using a 20X wide‐field objective (Nikon). The osteocyte density was evaluated as the number of DAPI‐positive cells within the bone matrix per mm^2^ of matrix area using the ImageJ software.

### Real‐time PCR analysis

Total RNA from MSC was extracted using RiboZol (Amresco, Solon, OH) following manufacturer's instructions. One *μ*g of RNA was reversed transcribed with AMV reverse transcriptase (Roche Diagnostics, Laval, Que., Canada) and the resulting complementary DNA was used for PCR on a MyiQ thermal cycler (BioRad, Mississauga, Ont., Canada). Real‐time relative quantification was performed using SYBR Green (BioRad). Primers specific for the genes of interest detailed in Table 1 were used, and the PCR were run for 40 cycles with an annealing temperature of 58°C for 30 sec. The expression of each gene was normalized to *B2m*, and then expressed as a null to WT ratio. The relative fluorescence units (RFUs) were analyzed with the iQ5 software (BioRad). The PCR products were visualized following electrophoresis on a 2% agarose gel stained with GoGreen (Invitrogen).

**Table 1.** Sequences of primers for gene expression.

Symbol	Primers	Access #
*B2m*	F: 5′‐TACTCACGCCACCCACCGGAG‐3′	NM_009735.3
	R: 5′‐GCTCGGCCATACTGGCATGCT‐3′	
*Lrp1*	F: 5′‐CTCCCACCGCTATGTGATCC‐3′	NM_008512.2
	R: 5′‐CACAGCTGTTGGTGTCGTTG‐3′	
*Lrp5*	F: 5′‐AAGGTTGTCGGAACCAACCC‐3′	NM_008513.3
	R: 5′‐CCTCGGGGATTATGCAGGTC‐3′	
*Lrp8*	F: 5′‐ACGTGCTCCAGTGAAGAGTG‐3′	NM_001080926.1
	R: 5′‐ACACTGAAATCTGCGGGGAC‐3′	
*Axin2*	F: 5′‐CCTGACCAAACAGACGACGA‐3′	NM_015732.4
	R: 5′‐CACCTCTGCTGCCACAAAAC‐3′	
*Lef1*	F: 5′‐TTCAAGGACGAAGGCGATCC‐3′	NM_010703.4
	R: 5′‐CTCTGGCCTTGTCGTGGTAG ‐3′	
*Scarb1*	F: 5′‐CAGCCTGACAAGTCGCATGGCTC‐3′	NM_016741.2
	R: 5′‐AAAAGCACGCTGGCCCATGGTG‐3′	
*CcnD1*	F: 5′‐CAAAATGCCAGAGGCGGATG‐3′	NM_007631.2
	R: 5′‐GAAAGTGCGTTGTGCGGTAG‐3′	
*CcnA2*	F: 5′‐ACCTGCCTTCACTCATTGCT‐3′	NM_009828.2
	R: 5′‐AGGTCTGGTGAAGGTCCACA‐3′	
*Col1a1*	F: 5′‐ACTTCAGCTTCCTGCCTCAG‐3′	NM_007742.3
	R: 5′‐GCTTCTTTTCCTGGGGTTC‐3′	
*Sp7*	F: 5′‐TTCGCATCTGAAAGCCCACT‐3′	NM_130458.3
	R: 5′‐TGCGCTGATGTTTGCTCAAG‐3′	
*Sost*	F: 5′‐CAGGAATGATGCCACAGAGGT‐3′	NM_024449.6
	R: 5′‐GTCTGTCAGGAAGCGGGTG‐3′	
*Runx2*	F: 5′‐CTCAGTGATTTAGGGCGCATTC‐3′	NM_001146038.2
	R: 5′‐TTAATAGCGTGCTGCCATTCG‐3′	
*Ocn*	F: 5′‐CAAGTCCCACACAGCAGCTT‐3′	NM_007541.3
	R: 5′‐AAAGCCGAGCTGCCAGAGTT‐3′	
*Dkk1*	F: 5′‐CGGTTCTTGGCCGTGTTTAC‐3′	NM_010051.3
	R: 5′‐GAGCAGTACTCGTCAGAGCC‐3′	
*Wnt5a*	F: 5′‐GTGATGCAAATAGGCAGCCG‐3′	NM_009524.3
	R: 5′‐AGCGTGGATTCGTTCCCTTT‐3′	
*Wnt3a*	F: 5′‐CCTCCGCTGGAGTAGCTTTC‐3′	NM_009522.2
	R: 5′‐GTTGTGACGGTTCATGGCAG‐3′	
*Rspo2*	F: 5′‐AGCGAATGGGGAACGTGTAG‐3′	NM_172815.3
	R: 5′‐CTTGCATCTCCTGGACTCCG‐3′	
*Dmp1*	F: 5′‐AGCTCAGAAAGCCAGTCCAC‐3′	NM_016779.2
	R: 5′‐TGGATCGGCTACTGTCCTGA‐3′	

### Statistical analysis

Statistical analyses were conducted with the Prism5 software (GraphPad, La Jolla, CA). Paired t tests, two‐way ANOVAs and Bonferroni post hoc tests, as indicated in the legend, were applied to determine statistical significance; a *P* value of 0.05 was considered as the significance threshold. All data are presented as mean ± SEM.

## Results

### Impact of SR‐BI deficiency on HDL_3_ association and binding, and OxLDL cytotoxicity in MSC

**Figure 1.**
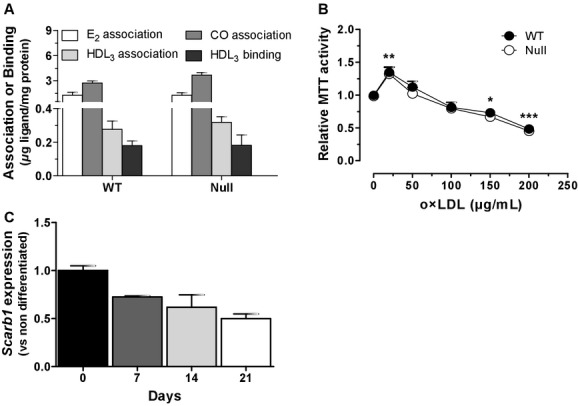
Lack of SR‐BI involvement in HDL association/binding and OxLDL‐induced cytotoxicity in mouse MSC. (A) Association of ^3^H‐E_2_‐HDL_3_ (E_2_ association), ^3^H‐CO‐HDL_3_ (CO association) and ^125^I‐HDL_3_ (HDL_3_ association), as well as ^125^I‐HDL_3_ binding (HDL_3_ binding) in WT and *Scarb1*‐null MSC measured following the procedures described in the Material and Methods. Data are average ± SEM of three to six independent cell preparations. **(**B) Metabolic activity measured by MTT assays in WT and *Scarb1*‐null MSC exposed to 0–200 *μ*g/mL of OxLDL for 48 h. Data are average ± SEM of four independent cell preparations. Bonferroni post‐hoc test with significant differences (*P <**0.05, **P <**0.01, ***P <**0.001) versus 0 *μ*g/mL. (C) *Scarb1* genic expression in primary MSC following osteogenic treatment for 21 days. Data are average ± SEM of three independent cell preparations. One‐way ANOVA with significant difference (*P *<**0.05).

Our previous results indicated that selective uptake of CE and estrogen from HDL_3_ was similar between WT and *Scarb1*‐null MSC (Martineau et al. 2014). Since SR‐BI is a high‐affinity receptor for HDL (Acton et al. 1996), we further investigated association and binding of HDL_3_ in MSC. As shown in Figure 1A, neither lipoprotein, lipid and estrogen association, nor lipoprotein binding were different between WT and *Scarb1*‐null MSC. Since SR‐BI has also been shown to bind and mediate internalization of OxLDL (Gillotte‐Taylor et al. 2001), we determined the OxLDL‐induced cytotoxicity in MSC from *Scarb1*‐null mice. Incubation of WT MSC with increasing OxLDL concentrations resulted in a dose‐dependent reduction in cell viability (Fig. 1B), agreeing with previous findings (Brodeur et al. 2008c; Hamel et al. 2008). Of interest, OxLDL induced similar cytotoxicity in *Scarb1*‐null MSC (Fig. 1B), suggesting that this receptor is not the main contributor to OxLDL‐induced cell death. Since absence of SR‐BI was associated to enhanced MSC functions (Martineau et al. 2014), *Scarb1* expression was monitored throughout osteogenic treatment to assess its role in osteoblasts. *Scarb1* gene expression was found to be down‐regulated with osteogenic differentiation (one‐way ANOVA, *P *<**0.05), hinting at a possible repressive role in MSC proliferation and/or osteoblastic differentiation (Fig. 1C).

### Contribution of HDL and SR‐BI to regulation of MSC proliferation and differentiation

**Figure 2.**
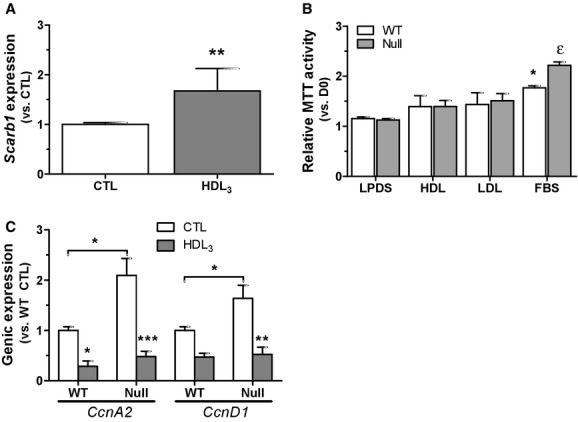
Effect of HDL on MSC of WT and *Scarb1*‐null mice. (A) *Scarb1* genic expression in WT MSC following 24 h exposure to HDL_3_ (150 *μ*g/mL). Data are average ± SEM of four independent cell preparations. Student *t*‐test with significant differences (*P *<**0.01) versus CTL. (B) Metabolic activity measured by MTT assays in WT and *Scarb1*‐null MSC cultured for 6 days in control medium (2% lipoprotein‐deficient serum‐LPDS) with or without 150 *μ*g/mL LDL or HDL, versus complete medium condition (10% FBS). Data are average ± SEM of six independent cell preparations. Bonferroni post‐hoc test with significant differences (*P <**0.05) versus LPDS condition or versus WT cells in FBS condition (_e_*P* < 0.05). (C) Gene expression of *CcnD1* and *CcnA2* in WT and *Scarb1*‐null MSC following a 24 h exposure to 150 *μ*g/mL of HDL_3_ or culture medium (CTL)_._ Data are average ± SEM of six independent cell preparations. Bonferroni post‐hoc test with significant differences versus WT or versus CTL (*P <**0.05, **P <**0.01, ***P <**0.001).

We recently reported enhanced proliferation of *Scarb1*‐null MSC (Martineau et al. 2014). Also, reports on the modulation of cell proliferation by HDL are numerous (Kothapalli et al. 2004; Yvan‐Charvet et al. 2007; Xu et al. 2012a). Since *Scarb1*‐null mice display increased HDL levels, we therefore investigated the effects of HDL and the involvement of SR‐BI in MSC proliferation. As shown in Figure 2A, *Scarb1* expression was increased by incubation of WT MSC with HDL_3_, a subclass of HDL rich in apoA‐I and binding more specifically to SR‐BI (Rigotti et al. 1997), which agrees with our previous results in MG‐63 osteoblast‐like cells (Brodeur et al. 2008b). However, neither the addition of HDL_3_ nor LDL significantly modified proliferation in WT or null cells (Fig. 2B), in spite of the greater proliferation in complete medium observed in the null cells. Accordingly, null MSC showed significantly higher gene expression of cell cycle regulators cyclin A2 (*CcnA2*) and cyclin D1 (*CcnD1*) under control conditions, yet HDL_3_ exposure reduced their expression in both WT and null cells (Fig. 2C).

### Impact of HDL_3_ on osteogenic markers in WT and Scarb1 null MSC

**Figure 3.**
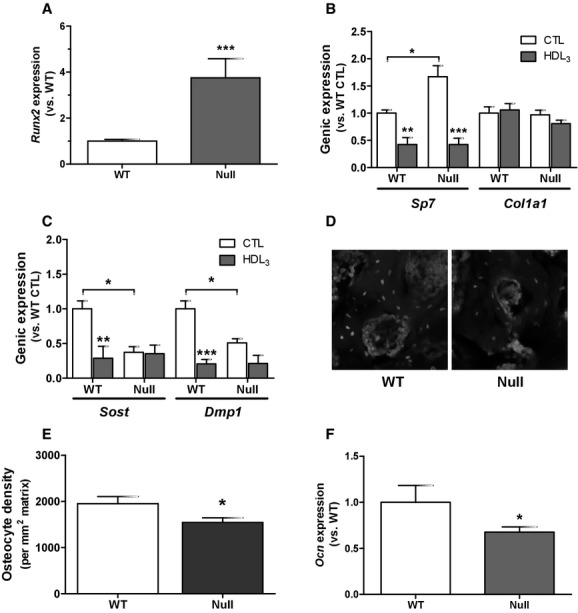
Expression of osteoblastic and osteocyte genes in WT and null cells. A) Gene expression of *Runx2* in WT and *Scarb1*‐null MSC under basal culture conditions. Data are average ± SEM of five independent cell preparations. Student *t*‐test with significant differences versus WT (***P <**0.001). (B,C) Gene expression of *Sp7* and *Col1a1* (B) as well as of *Sost* and *Dmp1* (C) in WT and *Scarb1*‐null MSC under basal conditions or following stimulation for 24 h with HDL (150 *μ*g/mL, B and C). Data are average ± SEM of three to six independent cell preparations. Bonferroni post‐hoc test with significant differences versus WT (*P < 0.05) or versus CTL (**P <**0.01, ***P <**0.001). (D) DAPI staining showing number of osteocytes, defined as, cells embedded within bone matrix, and (E) osteocyte density measured in WT and *Scarb1*‐null vertebrae. Data are from 11 mice per group. Student *t* test with significant differences (*P <**0.05) vs. WT. (F) Gene expression of *Ocn* in WT and *Scarb1*‐null MSC under basal culture condition. Data are average ± SEM of three independent cell preparations. Student *t*‐test with significant differences versus WT (*P <**0.05).

Because HDL_3_ did not promote proliferation in neither WT nor null MSC, we investigated whether these conditions would influence the expression of osteogenic markers. We previously reported that *Scarb1*‐null MSC show enhanced osteoblastic differentiation (Martineau et al. 2014). In accordance, *Runx2* and *Sp7* expression was increased in *Scarb1*‐null MSC under control culture conditions (Fig. 3A and B). Moreover, *Sp7* expression was significantly reduced by HDL_3_ exposure in both genotypes, whereas no effect was noticed on *Col1a1* expression (Fig. 3B). Both *Sost* and *Dmp1* osteocyte markers were significantly less expressed in the null cells (Fig. 3C); moreover, both *Sost* and *Dmp1* expression was reduced by HDL_3_ exposure in the WT cells, while no significant difference was observed in their expression in *Scarb1*‐null cells. Findings of reduced osteocyte marker expression in *Scarb1*‐null cells in vitro prompted us to measure the number of osteocytes in bone sections from *Scarb1*‐null mice. Nuclear DAPI staining of lumbar vertebrae sections for osteocytes, e.g. cells buried within bone matrix (Fig. 3D), showed lower osteocyte density in the null bones compared to WT bones (Fig. 3E), agreeing with the lower expression of osteocyte markers. Furthermore, *Scarb1*‐null cells express lower levels of *Ocn* mRNA (Fig. 3F).

### Enhanced canonical and noncanonical Wnt signaling in Scarb1‐null MSC

**Figure 4.**
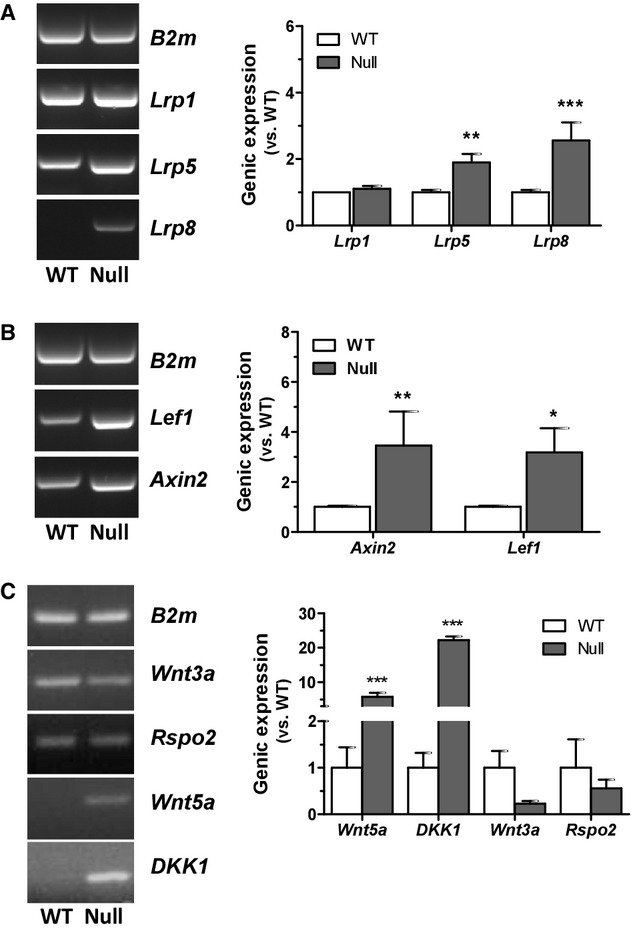
Expression of Wnt signaling pathway molecules in WT and *Scarb1*‐null MSC. Representative agarose gel electrophoresis of PCR products (left panels) and real‐time PCR analysis (right panels) for *Lrp1*,* Lrp5 and Lrp8* receptors (A), for Wnt pathway transcription‐targets *Axin2* and *Lef1* (B) and Wnt pathway ligands *Wnt5a*,* Dkk1*,* Wnt3* and *Rspo2* (C). Data are from four to six independent cell preparations. Bonferroni post‐hoc test with significant differences (*P <**0.05, **P <**0.01, ***P <**0.001) versus WT.

Since no clear correlation could be established between the in vitro cell behavior of *Scarb1*‐null MSC and either SR‐BI binding affinity or HDL effects in these cells, we investigated whether other pathways may be deregulated in the null cells. We previously measured lower *Cav1* expression in MSC from *Scarb1*‐null mice (Martineau et al. 2014). Since some components of the canonical Wnt signaling pathway have been localized in caveolae (Riddell et al. 2001; Yamamoto et al. 2006) and that this signaling pathway is involved in osteoblastogenesis (Boudin et al. 2013), we investigated potential alterations of the latter in *Scarb1*‐null MSC. A real‐time relative genic expression screening indicated overexpression of the *Lrp5* and *Lrp8,* while *Lrp1* remained unaffected (Fig. 4A). Moreover, *Lef1* and *Axin2*, known transcription‐targets of the Wnt signaling pathway, were also overexpressed in *Scarb1*‐null MSC (Fig. 4B). Further exploration also showed significant overexpression of the noncanonical pathway ligand *Wnt5a* and canonical pathway inhibitor *Dkk1* (Fig. 4C); despite a visible trend, the apparent reduction in *Wnt3a* and *Rspo2* expression was not statistically significant.

## Discussion

SR‐BI, the gene product of *Scarb1*, is an HDL receptor whose function in lipoprotein metabolism is well known. However, its role in peripheral tissues is unclear and does not always seem to correlate with its selective uptake functions. We previously reported higher bone mass in *Scarb1*‐null mice (Martineau et al. 2014). Though these mice show high HDL‐associated cholesterol levels (Rigotti et al. 1997; Martineau et al. 2014), whether their high plasma HDL‐associated cholesterol contribute to the enhanced bone formation remains unknown. On the other hand, intrinsic alterations of MSC functions from *Scarb1*‐null mice were evidenced in vitro, globally showing enhanced bone‐forming potential (Martineau et al. 2014). We report herein that *Scarb1* deficiency impacts neither HDL and associated lipids association or binding, nor MSC sensitivity to OxLDL. Secondly, HDL_3_ do not promote MSC proliferation and repress osteogenic marker expression in both genotypes. Finally, *Scarb1* deficiency seemingly stunts terminal osteocytic differentiation and alters genic expression of several Wnt signaling components.

Since SR‐BI is a high‐affinity receptor for HDL_3_, we verified whether some alterations in the association or binding capacities between WT and null cells occurred. No significant difference in lipid ligand or HDL_3_ association/binding was evidenced between WT and null MSC. These findings confirm our previous results showing unchanged levels of selective uptake of E_2_ and CO from HDL_3_ in WT and *Scarb1*‐null MSC (Martineau et al. 2014). These data state that SR‐BI is not the sole receptor involved in the selective uptake process in MSC and that other receptors are able to associate to lipid ligands and bind HDL_3_ in these cells. It is possible that the cluster of differentiation‐36 (CD36), a member of the SR‐B family, could rescue SR‐BI deficiency for HDL‐CE selective uptake as we have shown for LDL‐CE selective uptake in mouse liver (Luangrath et al. 2008). Moreover, other receptors such as LRP have been shown to participate in the selective uptake of lipids from HDL (Vassiliou et al. 2001).

We have previously reported OxLDL‐induced cytotoxicity in osteoblastic cells (Brodeur et al. 2008c; Hamel et al. 2008), yet the identity of the involved receptor(s) remains unknown. Since SR‐BI binds and internalizes OxLDL (Gillotte‐Taylor et al. 2001), we determined the OxLDL‐induced cytotoxicity in *Scarb1*‐null MSC. Oxidized LDL induced similar cytotoxicity in both genotypes, rendering a significant contribution of SR‐BI unlikely. Other receptors bind OxLDL and therefore, OxLDL‐induced cytotoxicity probably involves several different mechanisms independent of SR‐BI. Namely, the scavenger receptors class A (SR‐A) as well as CD36 are expressed by osteoblasts (Kalajzic et al. 2005; Brodeur et al. 2008a) and are known to participate in the internalization of OxLDL in macrophages, promoting their transformation into foam cells. Though being involved neither in HDL_3_ binding/association nor OxLDL‐induced cytotoxicity, *Scarb1* expression was significantly reduced in MSC undergoing osteogenic treatment; to assess whether this gene could be involved in HDL‐mediated regulation of cell functions, we further investigated proliferation and differentiation markers following HDL_3_ treatment.

Enhanced proliferation of *Scarb1*‐null MSC in control conditions was revealed by increased MTT activity in complete medium and augmented gene expression of *CcnA2* and *CcnD1*, which encode cyclins promoting cell cycle progression towards the S and G2/M phases, respectively (Meeran and Katiyar 2008). Of note, we reported that gene expression of *Cav1* is reduced in *Scarb1*‐null MSC (Martineau et al. 2014). Its gene product caveolin‐1 is known to repress transcription of *CcnD1* in smooth muscle cells and heart tissue (Hulit et al. 2000; Nagajyothi et al. 2006) and *CcnA2* in fibroblasts and smooth muscle cells (Kim et al. 2005); *Cav1* expression is down‐regulated by proliferative stimuli such as FGF or PDGF (Hulit et al. 2000). *Scarb1* therefore seems to be a negative regulator of proliferation by keeping MSC in G0/G1 quiescent phase, as SR‐BI has been linked to stabilization of caveolin‐1 expression (Frank et al. 2002), supporting our findings. Accordingly, enhanced proliferation was reported in *Cav1* KO mouse embryonic stem cells (Razani et al. 2001) and human MSC following *Cav1*‐targeted gene silencing (Baker et al. 2012). We next investigated whether HDL_3_ impact MSC proliferation. *Scarb1* expression is induced following HDL_3_ exposure as we previously reported in MG‐63 osteoblast‐like cells (Brodeur et al. 2008b); however, our data indicate that HDL_3_ has no effect on MSC proliferation as evidenced by MTT assays and lower mRNA levels of *CcnA2* and *CcnD1*. Moreover, the reduction in *CcnA2* and *CcnD1* expression was observed in both genotypes, suggesting an SR‐BI‐independent mechanism. These results diverge from the study of Xu et al. (Xu et al. 2012a) demonstrating that HDL‐induced proliferation of rat MSC was prevented by *Scarb1*‐targeted gene silencing. Discrepancy between these results and ours may arise from species differences and experimental procedures. In mice, HDL are reported to inhibit proliferation in hematopoietic stem/progenitor cells through an ATP‐binding cassette transporter A1 and G1 (ABCA1/G1)‐dependent mechanism (Feng et al. 2012), which may be at play in our model. Also, Xu et al. (Xu et al. 2012a) treated MSC with an HDL_2_/HDL_3_ mix in 2% FBS. We rather use HDL_3_, because of its greater selectivity towards SR‐BI binding (Rigotti et al. 1997), and lipoprotein‐deficient serum to avoid confounding effects from bovine lipoproteins present in serum. Moreover, a similar study reported that HDL did not promote rat MSC proliferation (Xu et al. 2012b).

Since HDL_3_ do not promote MSC proliferation in our system, we therefore questioned whether they influenced osteogenic marker expression. We have previously reported higher levels of *Sp7* yet normal levels of *Col1a1* mRNA in *Scarb1*‐null MSC (Martineau et al. 2014), which was confirmed in this study with the measurement of increased *Runx2* expression in *Scarb1*‐null MSC. Exposure to HDL_3_ had no impact on gene expression of *Col1a1* but reduced *Sp7* gene expression; this effect seemed independent of SR‐BI since observed in both WT and null cells. Interestingly, gene expression of *Sost* and *Dmp1* was reduced in *Scarb1*‐null MSC; accordingly, lower osteocyte density was observed in null mouse vertebrae. Osteocytes are expected to inhibit osteoblast proliferation and differentiation through secretion of SOST, encoded by the *Sost* gene (Komori 2013). Of interest, *Sost* overexpression in osteoblasts prevents load‐induced activation of Wnt signaling (Tu et al. 2012); moreover, disruption of *Cav1* expression in MLO‐Y4 osteocyte‐like cells reduces their mechanical response and survival (Gortazar et al. 2013). The reduction of *Sost* expression in *Scarb1*‐null MSC may be relevant, as its protein product is known to inhibit Wnt signaling and repress osteoblast proliferation and differentiation (Boudin et al. 2013; Komori 2013). Also, our data indicate that HDL_3_ reduced *Sost* and *Dmp1* expression in WT MSC; thus, though *Scarb1* deficiency itself promotes osteogenic differentiation of MSC, HDL_3_ treatment shows differential effects on osteogenesis by reducing *Sost*/*Dmp1* and *Sp7* simultaneously. Accordingly, we observed higher *Runx2* and lower *Ocn* expression in the null cells relatively to the WT cells; the transgenic overexpression of *Runx2*, isoform I, has been demonstrated to reduce *Ocn* gene transcription, yet no effect on *Col1a1* expression was observed (Kanatani et al. 2006), as we observed in our previous study (Martineau et al. 2014). Moreover, overexpression of either Runx2 isoforms reduces osteocyte density (Kanatani et al. 2006), similarly to what is observed in this study. However, Kanatani et al. (2006) also reported lower trabecular bone volume and cortical thickness in these mice, quite opposite to what our model displays; perhaps the degree of *Runx2* overexpression in *Scarb1*‐null mice, much lower than that in the *Runx2*‐transgenic line generated by Kanatani et al. (2006), is not sufficient to provoke detrimental effects on bone structure yet enough to induce some imbalance in cells from the osteoblastic lineage.

Because lower *Sost* expression in *Scarb1*‐null cells hinted at a possible alteration of Wnt signaling, we verified whether this pathway was affected. Several actors of canonical Wnt pathway were overexpressed in *Scarb1*‐null MSC, such as *Lrp5* and *Lrp8* which encode co‐receptors in Wnt signaling, as well as *Axin2* and *Lef1* that are transcription‐targets of this pathway. The Wnt pathway regulates proliferation and differentiation of osteoprecursors (Zhang 2012; Boudin et al. 2013) namely through upregulation of *CcnD1* gene expression by *Lef1,* leading to enhanced cell proliferation (Shtutman et al. 1999; Tu et al. 2012) and corroborating the higher expression of this gene in null cells. Since SOST is recognized to bind LRP5/6 and antagonize the canonical Wnt pathway (Li et al. 2005), its lower expression in *Scarb1*‐null MSC could promote Wnt signaling. Moreover, the *Lrp5* gene promoter contains *Runx2* response elements (Agueda et al. 2011), the latter promoting osteogenesis; perhaps the enhanced *Lrp5* expression in *Scarb1*‐null cells is linked to greater *Runx2* activity. Also, LRP8 was recently shown to be important in canonical Wnt signaling (Zhang 2012); its overexpression in *Scarb1*‐null cells also agrees with enhanced osteogenic potential. Moreover, we report that *Wnt5a* is overexpressed in *Scarb1*‐null MSC, suggesting overactivation of the noncanonical Wnt pathway as well. Considering that this pathway antagonizes canonical Wnt signaling in some systems (Davis et al. 2008), this may reflect a mechanism to re‐establish balance in the *Scarb1*‐null cells. The overexpression of *Dkk1* also speaks in that direction, since this factor prevents *β*‐catenin accumulation necessary for canonical Wnt signaling (Boudin et al. 2013). Paradoxically, high *Dkk1* osteoblast‐targeted overexpression is associated with lower bone mass (Yao et al. 2011). Nevertheless, patients with sclerostin deficiency (sclerosteosis and van Buchem disease) show increased bone formation despite significantly higher DKK1 plasma levels (van Lierop et al. 2014), which suggests an adaptative response to increased bone formation characterizing these diseases although high DKK1 levels do not compensate for the lack of sclerostin. Moreover, some rare metabolic diseases such as Schnitzler Syndrome are associated to high DKK1 plasma levels yet higher bone mass (Terpos et al. 2012). The reason why *Scarb1*‐null mice show greater osteoblastic surface (Martineau et al. 2014) yet fewer osteocytes remains speculative. Perhaps *Scarb1* deficiency disrupts the terminal osteocyte differentiation of MSC, keeping them in a “post‐osteoblast/pre‐osteocyte” state. Reduced osteocyte density along with lower *Sost* and *Dmp1* expression agree with the enhanced osteoblast surface in vivo and enhanced bone formation reported in *Scarb1*‐null mice (Martineau et al. 2014).

Herein we report that *Scarb1*‐null MSC spontaneously show greater proliferation and osteogenic differentiation, both stunted by HDL_3_ exposure. SR‐BI is likely a repressive regulator of osteogenesis as its expression is reduced in WT MSC following osteogenic treatment. The enhanced bone mass observed in *Scarb1*‐null mice is likely the combined effect of the previously reported high‐ACTH levels (Martineau et al. 2014) and intrinsic cellular alterations of the canonical and noncanonical Wnt pathways, and poorly related to plasma HDL levels in *Scarb1*‐deficient mice. Future studies will focus on the potential crosstalk between SR‐BI and both canonical and noncanonical Wnt pathways.

## Conflicts of interest

None declared.
